# B7-H3 in the tumor microenvironment: Implications for CAR T cell therapy in pediatric solid tumors

**DOI:** 10.1007/s10555-025-10294-y

**Published:** 2025-10-11

**Authors:** Lena Jansen, Judith Wienke, Ronja Molkenbur, Claudia Rossig, Ramona Meissner

**Affiliations:** 1 Princess Máxima Center for Pediatric Oncology, Heidelberglaan 25, 3584 CS Utrecht, The Netherlands; 2 Department of Pediatric Hematology and Oncology, University Children’s Hospital Muenster, Muenster, Germany

**Keywords:** CAR T cells, B7-H3, CD276, Immune checkpoints, Tumor microenvironment, Pediatric solid cancers

## Abstract

B7 homolog 3 (B7-H3, CD276) has emerged as a promising target for chimeric antigen receptor (CAR) T cell therapy, with limited expression in normal tissues and high level cell-surface expression across various tumor types. Clinical studies are ongoing, with a focus on pediatric cancers. As an immune checkpoint molecule of the B7-CD28 family, B7-H3 has a proposed immune-modulatory role, though the precise nature of B7-H3-mediated cell interactions and functional contributions to immune responses are contradictory and likely context-dependent. Within tumors, B7-H3 is expressed also on non-tumor cell types in the tumor microenvironment (TME), including myeloid immune cells, endothelial cells of abnormal vasculature and cancer-associated fibroblasts. Consequently, CAR T cells directed against B7-H3 will not only target tumor cells but also components of the TME, which will affect the nature and outcome of B7-H3-targeted therapeutic immune responses. Here we review the expression of B7-H3 protein in pediatric solid tumors and in various cell types known to infiltrate the TME of solid tumors. On this background, we discuss the potential of B7-H3-targeted CAR T cells to reshape the TME and the key challenges and future directions to improve B7-H3-targeted CAR T cell therapy for pediatric patients with solid cancers.

## Introduction

The development of effective therapies for pediatric solid cancers based on antiproliferative chemotherapies combined with local tumor treatments has led to high probabilities of event-free survival [1]. But the survival curves have reached plateaus, and all established treatments are flawed by significant toxicities and late effects [2, 3]. To achieve further progress, novel therapies that act by alternative mechanisms are urgently needed.

Cellular immunotherapy with T cells engineered to express chimeric antigen receptors (CARs) is emerging as a transformative approach for cancer treatment. Unlike conventional T cell receptors, CARs activate T cells via surface-expressed molecules, independent of peptide presentation, enabling potent antitumor response [4]. Upon recognizing their target, CAR T cells expand *in vivo*, eliminate large numbers of cancer cells, and can persist as memory T cells to prevent relapse [5].

The most striking success has been achieved in B cell malignancies. CAR T cells targeting B-lineage antigens have induced durable remissions in adults with lymphoma [6] and in children with refractory B-lineage acute lymphoblastic leukemia (B-ALL) [5]. These results demonstrate the ability of CAR T cells to eradicate even large disease burden across various anatomical sites [5]. In contrast, effective CAR T cell therapy of solid tumors remains a challenge. Early trials targeting antigens such as ganglioside GD2 [7–9], GPC3 [10], Claudin-6 [11], Claudin-18.2 [12] and B7-H3 [13] have reported objective responses, but overall efficacy in extracranial solid and central nervous system (CNS) cancers has been modest. Major barriers include heterogeneous antigen expression, provoking antigen-negative escape, and active immunosuppression by the tumor microenvironment (TME) which restricts T cell infiltration, function and persistence [14].

An attractive cell surface target in solid cancers is B7 homolog 3 (B7-H3), also known as CD276. This transmembrane glycoprotein belongs to the B7 family of immune checkpoint molecules and was originally identified through antibody screens in neuroblastoma, the most common extracranial solid tumor in children [15]. B7-H3 was found to be expressed across a broad range of solid tumors both in adults (outside the scope of this review) and in the pediatric population, with limited protein expression in healthy tissues [15–21]. This selective expression makes B7-H3 an attractive, widely applicable target for CAR T cells and other cell-surface directed therapeutics. Various clinical trials are investigating B7-H3-targeted therapies in both adult and pediatric cancers. These studies employ a range of strategies, besides CAR T cells also non-cellular therapies such as monoclonal antibodies [22, 23], radioimmunoconjugates [24, 25] and antibody–drug conjugates (ADCs) [26, 27]. Two early clinical trials have used B7-H3-targeted CAR T cells in pediatric cancer populations, both with a second generation CAR T cell product with integrated 4-1BB co-stimulation. One of these studies treated patients with diffuse midline glioma (DMG), a cancer of the CNS (NCT04185038) [28, 29]. Repeated locoregional (i.e., intraventricular) infusions of B7-H3 CAR T cells were safe and induced clinical responses, including a partial remission. A second trial with systemic administration of CAR T cells to 9 pediatric and young adult patients with various extracranial solid cancers (NCT04483778) showed limited overall activity. One patient achieved a partial remission with substantial expansion of CAR T cells after a second dose, however, dose-limiting liver toxicity was reported [13]. While various studies with B7-H3-targeting CAR T cell products are ongoing (e.g., NCT04897321, NCT05835687, NCT0576880), reported antitumor activity of B7-H3-specific CAR T cell therapy remains limited. Local mechanisms of immune suppression likely prevent effective interactions between CAR T cells and tumor cells.

A notable feature of the cancer target B7-H3 is its nature as an immune regulator, with still incompletely understood roles in physiological immune responses and within the TME. Expression on both tumor and stromal cells implies that B7-H3 targeting could modulate the composition of the TME in various ways. This could have a substantial impact on CAR T-cell induced local immune interactions, enhancing or dampening therapeutic immune responses. To inform the development of more effective B7-H3-targeting CAR T cell strategies and fully exploit the potential of this target, particularly in pediatric cancers, this review will provide a comprehensive overview of B7-H3 expression patterns, focusing on protein expression in biopsies of pediatric solid tumors. In addition, we will summarize existing knowledge on B7-H3 protein expression across the various non-malignant cell populations found in solid tumors and discuss the respective ways in which B7-H3-targeted CAR T cell therapy can be expected to reshape the TME.

## Physiological expression and functions of B7-H3

B7-H3 by structure is a type 1 transmembrane glycoprotein of the immunoglobulin (Ig) superfamily with two known isoforms in humans. The isoforms are distinguished by their extracellular motif composed of either one variable (IgV) and one constant (IgC) domain (2Ig-B7-H3) or two IgV-IgC domains (4Ig-B7-H3) [30]. While both isoforms are found intracellularly, the predominant isoform expressed on the surface of human cells is 4Ig-B7-H3 [30]. A soluble form is generated by alternative splicing [31] or, more commonly, via proteolytic cleavage by membrane metalloproteinases [32]. Soluble B7-H3 (sB7-H3) is detectable in human blood during bacterial infections and is considered a biomarker for meningitis and pulmonary infection in children [33, 34]. In line with a physiological role in immune regulation, B7-H3 protein is found on the surface of various immune cells in a context-dependent, activation-inducible manner [35]. This includes dendritic cells (DCs), monocytes and tissue-resident myeloid cell populations, and NK cells. Inflammatory *in vitro* conditions, e.g. stimulation of blood-derived DCs with IFN-γ or PMA (phorbol 12-myristate 13-acetate) and ionomycin induces upregulation of B7-H3 protein [36]. And while membrane-bound B7-H3 is absent on peripheral T cells under non-inflammatory conditions, elevated levels of soluble B7-H3 were detected in the cell culture supernatant after stimulation of T cells with IFN-γ or CD3/CD28 monoclonal antibodies [32].

The physiological function of B7-H3 in immune cell activation remains controversial, with both stimulatory and inhibitory roles reported [37]. The original study reporting identification of human B7-H3 had found that B7-H3-positive DCs can act as positive co-stimulators, enhancing IFN-γ production during T-cell activation [36]. Subsequent research by others has identified potent inhibitory effects of B7-H3 on T cells, limiting proliferative response as well as cytokine production of both naïve and activated T cells, by a mechanism involving suppression of IL-2 signaling [38]. Moreover, T cells with a regulatory phenotype (CD4^+^CD25^+^) were found to induce B7-H3 expression on DCs while simultaneously downregulating MHC-peptide complexes, with an overall suppressive effect on T cell activation and T-cell-mediated immune responses [39]. In cancer, high B7-H3 expression on tumor cells was typically associated with reduced infiltration by NK cells and CD8^+^ T cells, indirectly pointing towards a role as inhibitory immune checkpoint that facilitates immune evasion [40, 41]. Direct evidence was generated in a mouse model, in which B7-H3 deficiency or treatment with an antagonistic antibody enhanced antitumor immune responses mediated by NK cells and CD8^+^ T cells [41].

One hypothesis to explain conflicting data regarding the function of B7-H3 in T-cell mediated immune responses is its potential interaction with multiple receptors, resulting in different functional outcomes. Several candidate receptors for B7-H3 have been suggested. Trigger receptor expressed on myeloid cells-like transcript 2 (TLT-2) was suggested to act as putative B7-H3 receptor [42, 43] but this was not reproduced by others [38, 44]. More recently, interleukin-20 receptor subunit α (IL-20Rα) [45, 46] and phospholipase A2 receptor 1 (PLA2R) [46] were suggested to bind B7-H3 ligand. An additional explanation could be that membrane bound and soluble forms of B7-H3 may have diverse functions.

Beyond its roles in immune regulation, B7-H3 is involved in a wide variety of physiological processes. These include cell differentiation, of osteoblasts during embryogenesis [47, 48] and of mesenchymal stromal cells towards adipocytes [49]. In experimental tumor models, the upregulation of B7-H3 has also been associated with multiple features of cancer, e.g. promotion of malignant cell proliferation, invasion, migration and metastasis, angiogenesis, metabolic reprogramming and resistance to chemotherapy [50–52]. Various studies found expression of B7-H3 in tumors to be associated with poor prognosis [17, 19, 53–55].

## Regulation of B7-H3 protein expression

**Fig. 1:**
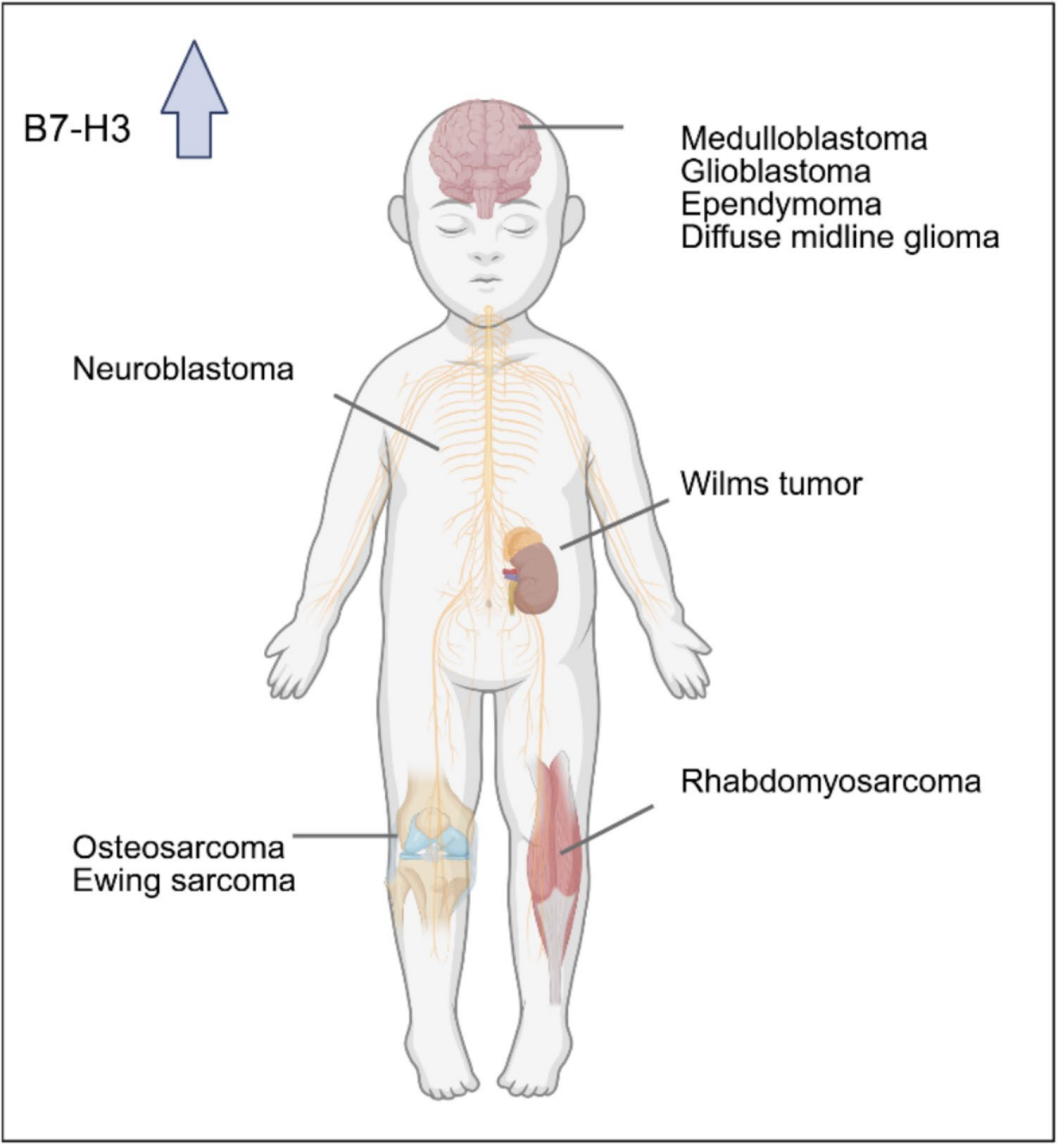
B7-H3 is a cell surface target with wide expression across pediatric solid tumors and tumors of the central nervous system (CNS)

Most pediatric solid tumors show upregulation of B7-H3 protein on the tumor cell surface (Fig. 1). However, expression levels vary widely among patients and among tumors, which can also lack B7-H3 protein. To be eligible for CAR T cell therapy, expression of the target antigen on the tumor is indispensable and the antigen density will most likely impact the treatment outcome. Tools have been developed that allow reliable assessment of cell surface expression of the antigen in tumor tissues from pediatric patients who are candidates for clinical trials with B7-H3 targeting agents [21, 56].

A notable feature of B7-H3 expression that complicates the investigation is that the presence of B7-H3 mRNA does not correlate with the presence of B7-H3 surface protein, which is indispensable for effective CAR T cell therapy. Therefore, gene expression studies are inadequate to inform about the targetability of tumors with B7-H3 directed immunotherapeutics nor about expression on healthy tissues that could result in relevant off-tumor toxicities. For example, Chapoval *et al.* detected B7-H3 mRNA in normal human (adult) tissues such as heart, kidney and lung [36], while B7-H3 protein was absent on these tissues [57, 58]. Another study showed ubiquitous expression of B7-H3 gene transcripts in fetal tissues, however, the authors of this study did not analyze B7-H3 protein expression [59]. Low levels of B7-H3 protein have been detected in various tissues, including prostate, testis, pancreas, liver, colon and appendix, stomach, placenta, pituitary, salivary and adrenal glands, breast and adipose tissue all of adult origin [57, 60]. The B7-H3 expression levels in these tissues are considered to be below the threshold required for CAR T cell activation [58], creating a therapeutic window for CAR T cell therapy. In the first published study of systemic use of B7-H3-specific CAR T cells in pediatric patients, dose-limiting liver toxicity was observed in a single patient with a tumor response after a second dose leading to high CAR T cell expansion in blood [13]. More clinical experience will be needed to understand to what extent the low B7-H3 protein expression on liver and other healthy tissues can cause clinical on-target/off-tumor toxicities and impair the safety profile of B7-H3-targeted therapies. The concept of logic-gated T-cell engineering could be a means to circumvent off-tumor toxicities. AND-gated CAR T cells rely on activation stimuli from two different antigens for full functionality, which can enable tumor-selective targeting of CAR T cells and avoid cross-reactivity with normal tissues [61, 62].

**Fig. 2:**
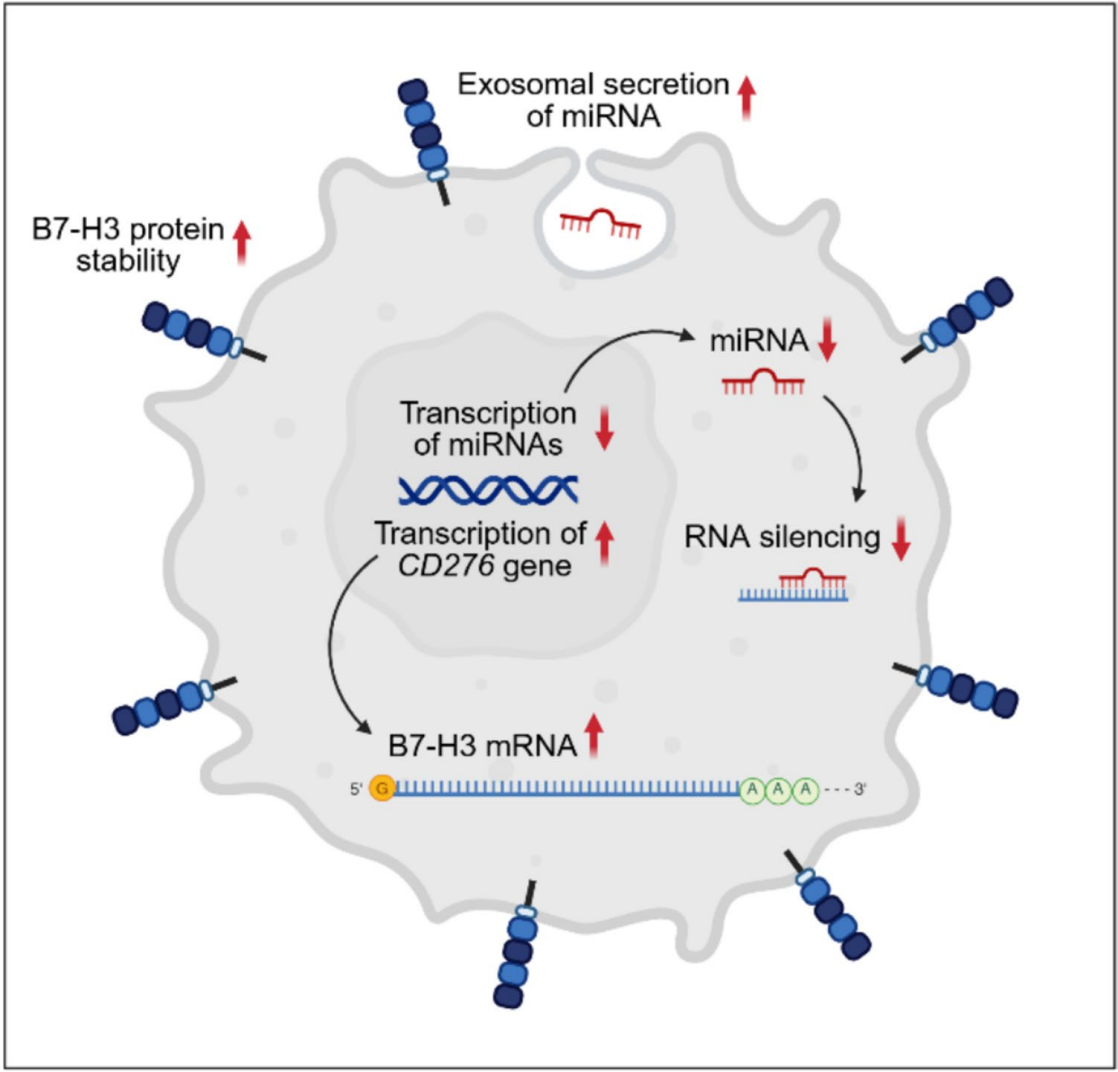
Various mechanisms on both transcriptional and translational level regulate the expression of B7-H3 in normal cells. Cancer cells have evolved strategies to upregulate B7-H3 protein, for example by downregulation of miRNAs

An explanation for the heterogenous expression of B7-H3 among tumors and the inconsistency between B7-H3 RNA and surface expression could be the diverse regulatory mechanisms that control B7-H3 protein expression [63, 64] (Fig. 2). Transcription of the *CD276* gene encoding B7-H3 has been shown to be upregulated in adult cancer types through epigenetic dysregulation, such as acetylation of the histones H3K27 in prostate cancer [65] or H4K16 in nasopharyngeal carcinoma [66]. Another mechanism that can upregulate *CD276* transcription is the activation of intracellular signaling pathways, such as p38 MAPK-eIF4E [67], mTORC1 [68] and PAX3-FOXO1 [69]. The precise mechanisms underlying posttranscriptional regulation of B7-H3 protein expression are unresolved [37], but increasing evidence suggests that small non-coding RNAs, microRNAs (miRNAs), regulate B7-H3 protein expression both in non-malignant and malignant cells [63, 64, 70]. MiRNAs bind to target mRNAs and initiate their degradation, thus reducing or abrogating protein expression. In many tumor cells, miRNAs that target B7-H3 mRNA were found to be downregulated, which reduces mRNA degradation and leads to elevated B7-H3 protein expression [63, 64]. Mechanisms by which tumor cells decrease the amount of individual miRNAs are reduced transcription or exosomal secretion. Candidate miRNA regulators for B7-H3 are miR-29 [63], which is downregulated in a variety of solid tumors [63, 70] and inversely associated with B7-H3 protein expression in neuroblastoma [63], and miR-124, downregulated and inversely correlated with B7-H3 protein expression in osteosarcoma [64]. In medulloblastoma, a pediatric brain cancer, histone methyltransferase enhancer of zeste homolog 2 (EZH2) was found to be overexpressed and involved in post-transcriptional upregulation of B7-H3 protein expression via downregulation of miR-29 [71]. As additional mechanism, studies in breast cancer [72] and neuroblastoma [73] have found inhibition of ubiquitin-mediated degradation of B7-H3 in the proteasome, resulting in increased stability and surface expression of the protein on tumor cells.

The inconsistency between mRNA and protein levels raises caution for the interpretation of B7-H3 expression data that rely on transcription levels alone. Comprehensive gene expression data recently reported for many pediatric cancers, including single cell analyses of tumor cells in their microenvironment, cannot adequately predict the targetability of individual cancers via B7-H3 CAR T cells. Flow cytometry analysis of B7-H3 surface expression on single cell suspensions of tumor cells most accurately quantifies surface expression but is limited to the analysis of cell lines or fresh tumor cell suspensions, which are not broadly available. Immunohistochemistry (IHC) analysis of formalin-fixed paraffine-embedded (FFPE) or cryopreserved tumor tissues adequately addresses B7-H3 protein but cannot easily differentiate between single individual cells. Despite these limitations, based on the availability of material, IHC analysis of FFPE tumor biopsies with B7-H3-specific antibodies is the most broadly applicable assay to stratify patients for B7-H3-targeted therapy [21, 56].

## Expression of B7-H3 in pediatric solid tumors

We conducted a comprehensive literature review on B7-H3 protein expression across various pediatric solid tumors (Table 1), including studies solely based on IHC. Studies that include specimens from adult patients (up to the age of 38 years) are marked. B7-H3 expression varied widely among individual studies. For the most frequent extracranial solid tumors of childhood and adolescence, the reported data were most consistent for neuroblastoma [53, 58, 60, 74, 75], rhabdomyosarcoma [40, 53, 58, 60, 74] and Wilms tumor [53, 74], with B7-H3 expression in 55–97%, 67–100% and 88–100%, respectively. More variable data were reported for Ewing sarcoma [53, 58, 60, 74], with B7-H3 expression ranging from none to 100% of samples, and for osteosarcoma, with 28–97% [17, 60, 74, 76, 77]. A high B7-H3 positivity rate was found in pediatric CNS tumors such as Medulloblastoma, high grade glioma (HGG) and diffuse midline glioma (DMG) with 75–100% [21, 53, 56, 58, 78, 79], 84–100% [21, 56, 58, 78] and 80–100% [21, 56, 58, 78, 80], respectively. Ependymoma showed a higher variability in B7-H3 expression ranging from 55–100% [21, 56, 78, 81, 82]. For atypical teratoid rhabdoid tumor (ATRT), a rare cancer of early childhood, either none or 100% of samples were B7-H3-positive [21, 56, 78, 82]. Biological explanations for these discrepant results include heterogeneity of B7-H3 protein expression and the various regulatory mechanisms. Indeed, substantial spatial and inter- and intratumoral heterogeneity in B7-H3 expression was found, raising concerns about potential escape of B7-H3-low or -negative tumor cells from CAR T cell recognition. B7-H3 expression may vary also with biological variability in subtypes of the same cancers and along disease progression. In addition, sampling and technical issues could have affected the results. Studies included pre- and post-treatment biopsies, not acknowledging potential effects of therapy on B7-H3 expression. Different antibodies with varying affinity and specificity and different tissue fixation and/or antigen retrieval methods, affecting antigen preservation and epitope recognition, were used among investigators. Noteworthy, different cutoff values for the H-score, commonly used in histology to determine positivity of stained samples, could lead to under- or overestimation of B7-H3 positive samples. Finally, subjective interpretation of the semi-quantitative scoring methods used in IHC analysis complicates comparison of results.

**Table 1:** B7-H3 protein expression across pediatric solid tumors

Cancer type	Sample number	Tissue	Antibody (clone), source	Positivity rate	Ref
Extracranial solid tumors					
Neuroblastoma	*n* = 18	FFPE	Rabbit mAb (EPNCIR122), Abcam	94%	[75]
	*n* = 20	FFPE	Rabbit mAb (D9M2L), Cell Signaling Technology	55%	[60]
	*n* = 186	FFPE	Goat polyclonal Ab, R&D Systems	82%	[58]
	*n* = 53	FFPE	Mouse mAb (5B14), generated by Castriconi *et al.* [15]	74%	[53]
	*n* = 90	Fresh frozen	Mouse mAb (8H9), generated by Modak *et al* [74]	97%	[74]
Ganglioneuroblastoma	*n* = 25	FFPE	Goat polyclonal Ab, R&D Systems	60%	[58]
Ganglioneuroma	*n* = 11	FFPE	Goat polyclonal Ab, R&D Systems	36%	[58]
Osteosarcoma	*n* = 15	FFPE	Rabbit mAb (D9M2L), Cell Signaling Technology	80%	[60]
	*n* = 32	FFPE	Rabbit mAb (D9M2L), Cell Signaling Technology	53%	[77]^a^
	*n* = 32	FFPE	Rabbit mAb (EPR20115), Abcam	28%	[76]^b^
	*n* = 29	Fresh frozen	Mouse mAb (8H9), generated by Modak *et al.* [74]	97%	[74]
Ewing sarcoma	*n* = 20	FFPE	Rabbit mAb (D9M2L), Cell Signaling Technology	0%	[60]
	*n* = 6	FFPE	Mouse mAb (5B14), generated by Castriconi *et al.* [15]	33%	[53]
	*n* = 27	FFPE	Goat polyclonal Ab, R&D Systems	89%	[58]
	*n* = 21	Fresh frozen	Mouse mAb (8H9), generated by Modak *et al.* [74]	100%	[74]
Rhabdomyosarcoma (alveolar and embryonal type not differentiated)	*n* = 9	FFPE	Mouse mAb (5B14), generated by Castriconi *et al.* [15]	67%	[53]
	*n* = 132	FFPE	Goat polyclonal Ab, R&D Systems	92%	[40]
	*n* = 29	Fresh frozen	Mouse mAb (8H9), generated by Modak *et al.* [74]	97%	[74]
Embryonal rhabdomyosarcoma	*n* = 10	FFPE	Rabbit mAb (D9M2L), Cell Signaling Technology	70%	[60]
	*n* = 10	FFPE	Goat polyclonal Ab, R&D Systems	100%	[58]
Alveolar rhabdomyosarcoma	*n* = 12	FFPE	Goat polyclonal Ab, R&D Systems	92%	[58]
Desmoplastic small round cell tumor	*n* = 11	FFPE	Rabbit mAb (D9M2L), Cell Signaling Technology	73%	[60]
Wilms tumor	*n* = 12	FFPE	Mouse mAb (5B14), generated by Castriconi *et al.* [15]	100%	[53]
	*n* = 8	Fresh frozen	Mouse mAb (8H9), generated by Modak *et al.* [74]	88%	[74]
Malignant peripheral nerve sheath tumor	*n* = 9	FFPE	Rabbit mAb (D9M2L), Cell Signaling Technology	67%	[60]
Tumors of the CNS					
Medulloblastoma	*n* = 46	FFPE	Goat polyclonal Ab, R&D Systems	96%	[58]
	*n* = 7	FFPE	Mouse mAb (5B14), generated by Castriconi *et al.* [15]	100%	[53]
	*n* = 33	FFPE	Rabbit mAb (SP206), Abcam	100%	[78]
	*n *= 24	FFPE	Rabbit polyclonal Ab, Novus Biologicals	96%	[79]
	*n* = 31	FFPE	Rabbit mAb (D9M2L), Cell Signaling Technology	97%	[56]
	*n* = 8	FFPE	Rabbit mAb (D9M2L), Cell Signaling Technology	75%	[21]
Ependymoma	*n* = 44	FFPE	Rabbit mAb (D9M2L), Cell Signaling Technology	66%	[81]
	*n* = 24	FFPE	Rabbit mAb (SP206), Abcam	100%	[78]
	*n* = 1	FFPE	Rabbit mAb (D9M2L), Cell Signaling Technology	100%	[21]
	*n* = 44	FFPE	Mouse mAb (F-11), Santa Cruz	55%	[82]
	*n* = 30	FFPE	Rabbit mAb (D9M2L), Cell Signaling Technology	70%	[56]
High grade glioma (HGG)	*n* = 37	FFPE	Goat polyclonal Ab, R&D Systems	84%	[58]
	*n* = 6	FFPE	Rabbit mAb (D9M2L), Cell Signaling Technology	100%	[21]
	*n* = 29	FFPE	Rabbit mAb (D9M2L), Cell Signaling Technology	93%	[56]
	*n* = 15	FFPE	Rabbit mAb (SP206), Abcam	100%	[78]
Diffuse midline glioma (DMG)	*n* = 29	FFPE	Rabbit mAb (D9M2L), Cell Signaling Technology	80%	[56]
	*n* = 21	FFPE	Rabbit mAb (SP206), Abcam	95%	[78]
	*n* = 22	FFPE	Goat polyclonal Ab, R&D Systems	100%	[58]
	*n* = 2	FFPE	Rabbit mAb (D9M2L), Cell Signaling Technology	100%	[21]
	*n* = 9	FFPE	Goat polyclonal Ab, R&D Systems	100%	[80]
Atypical teratoid rhabdoid tumor (ATRT)	*n* = 13	FFPE	Rabbit mAb (SP206), Abcam	100%	[78]
	*n* = 2	FFPE	Rabbit mAb (D9M2L), Cell Signaling Technology	0%	[21]
	*n* = 9	FFPE	Mouse mAb (F-11), Santa Cruz	100%	[82]
	*n* = 9	FFPE	Rabbit mAb (D9M2L), Cell Signaling Technology	100%	[56]

^a^Includes samples from both pediatric and adult patients (age 5–38 years)

^b^Includes samples from both pediatric and adult patients (age up to 35 years)

Available studies do not comprehensively address B7-H3 expression in individual cancers at different stages of disease or include comparative and longitudinal analyses between first diagnosis versus relapse and between primary tumors versus metastatic manifestations. A recent study detected B7-H3 in bone marrow aspirates of neuroblastoma patients and confirmed maintained B7-H3 expression at relapse, whereas expression of an alternative surface target, GD2, significantly declined with disease progression [83]. Other investigators found B7-H3 expression in neuroblastoma to be consistent across both adrenergic and mesenchymal cell lines, contrasting with other immunotherapeutic targets that exhibit more selective expression patterns in neuroblastoma [84]. Together, these findings confirm the consistency of B7-H3 expression in neuroblastoma and thus support its usefulness as therapeutic target. Results in other solid cancers are less stringent. Knockdown of the rhabdomyosarcoma-specific fusion gene *PAX3-FOXO1* was found to result in a decrease in B7-H3 expression, suggesting a direct link between the disease-causing translocation and regulation of B7-H3 expression in alveolar, translocation-positive rhabdomyosarcoma [69]. Comparisons between fusion-positive and -negative rhabdomyosarcoma samples, on the other hand, did not find a significant difference in B7-H3 expression [40]. Irrespective of the potential of B7-H3 as a pan-cancer antigen [58], our data collection reveals very heterogenous expression between tumor entities and individuals of the same tumor type (Table 1).

The presence of mRNA alone does not reveal the level of surface protein expression, which is a crucial determinant for CAR T cell therapy. Individual screening of tumor material before treatment using a stringent, standardized detection assay is desired to identify the patients who are most likely to benefit from B7-H3-targeted CAR T cell therapy. Furthermore, broad use of a harmonized assay is essential to understand B7-H3 expression in relation to tumor-specific mutations and to various manifestations and stages of disease across cancer types. Repeated analysis of tumor material during and after CAR T cell therapy, though limited by the need for re-biopsies, will help to understand the relevance of B7-H3-negative tumor cell escape.

## B7-H3 expression on non-cancer cell populations in the TME

For efficient eradication of solid tumors, the nature of the TME must be considered. A dynamic and complex network of immune and stromal cells, aberrant vasculature and a disorganized extracellular matrix (ECM) supports tumor cell survival, growth and metastasis and excludes or functionally inactivates antitumor effector cells. There is increasing evidence that tumor cells shape their own niche, e.g. by the release of chemokines or other soluble factors that preferentially recruit tumor-supporting immune cells. These include myeloid-derived suppressor cells (MDSCs), M2-polarized tumor-associated macrophages (TAMs) and regulatory T cells (Tregs). Immune-suppressive cell populations in the TME secrete type II cytokines (such as IL-4, IL-10, and IL-13), attract additional suppressive cell components of the immune system and induce T-cell exhaustion via checkpoint ligands [85].

In addition to upregulation in many solid tumors, B7-H3 protein can be expressed in the various cell types that compose the TME [49]. Consequently, B7-H3-targeted CAR T cells will likely eliminate non-malignant cell populations with immune-modulating function and thereby reshape the local TME. The modulation of the TME could substantially affect the crosstalk between tumor cells and immune effector cells and thus the outcome of therapeutic antitumor immune responses. Here we review current knowledge on the contribution of B7-H3-expressing non-tumor cell populations to the TME. To date, information is largely limited to material from adult cancers and to mouse models, with scarce insights into the specifics of pediatric solid tumors. At the same time, the predominance and relevance of individual cell infiltrates in the TME varies substantially in different cancer contexts [86, 87], thus translation of knowledge obtained in studies of adult cancers to pediatric tumors requires caution. Still, understanding the expression of B7-H3 on cell types commonly found in solid tumors overall could inform purposeful research in pediatric cancers and ultimately support the development of B7-H3 targeted CAR T cell therapeutics. To ensure relevance to cell-surface targeted immunotherapies, we exclusively summarize data from B7-H3 protein analyses and exclude data based on mRNA expression alone.

**Myeloid cell populations:** Among myeloid cells present in solid tumors, key populations are dendritic cells (DCs), tumor-associated macrophages (TAMs), myeloid-derived suppressor cells (MDSCs) and neutrophils. The presence of **DCs** in tumors can indicate presentation of tumor-associated antigens to T cells and thus an active contribution to antitumor immunity, but immunosuppressive subpopulations and functions have also been described [88]. In a study in non-small cell lung cancer (NSCLC), tumor-residing DCs were found to significantly upregulate B7-H3 compared to their counterparts in non-malignant lung tissue [89]. B7-H3-positive DCs in tumors were characterized by high secretion of the immunosuppressive cytokine IL-10 and reduced production of immune-activating IL-12 and were associated with decreased T cell proliferation in mixed lymphocyte reactions. In hepatocellular cancer (HCC), significantly higher levels of B7-H3 were detected on DCs in the tumor tissue compared to peripheral blood, normal liver tissue and surrounding para-tumoral regions [41]. Tumor-infiltrating DCs expressing B7-H3 were less potent in the induction of CD8 + T cell and NK cell responses. Blockade of B7-H3, also identified on further myeloid cell populations in the TME of HCCs in this paper, generated effective antitumor immune responses in a syngeneic mouse model [41]. Thus, B7-H3 could mark an immunosuppressive population of DCs in human tumors and/or contribute to their immunomodulatory function.

**TAMs** play a pivotal role in shaping the TME. Often polarized toward a pro-tumor M2-like phenotype, TAMs contribute to various processes that facilitate tumor progression, including angiogenesis, ECM remodeling and suppression of anti-tumor immune responses [90, 91]. TAMs with M2-like phenotype are a well-recognized cell population in the TME also of pediatric cancers, including neuroblastoma [92–95], Ewing sarcoma [96–98], rhabdomyosarcoma [99] and osteosarcoma [100, 101], with substantial functional evidence for a key role in promoting tumor growth and immune escape. Studies on B7-H3 expression on TAMs are largely limited to adult cancers where wide expression was found across different cancer types and elevated expression levels correlated with enhanced immunosuppressive and pro-tumorigenic functions [102]. In pancreatic cancer, for instance, higher B7-H3 expression on CD68-positive TAMs was found to be a predictor of poor prognosis [103]. In triple-negative breast cancer (TNBC), B7-H3 was enriched on TAMs in both patient-derived material and in mouse models [104]. B7-H3-expressing TAMs were not only immunosuppressive but also contributed to the remodeling of the ECM and to a pathological tumor vasculature, processes that impede T cell infiltration into the TME and facilitate tumor progression [104]. In non-small cell lung cancer (NSCLC), B7-H3 protein was detected on macrophages in tumor tissue at substantially higher levels compared to non-neoplastic tissues [105]. And in patients with HCC, B7-H3 protein expression was found to be elevated on CD14 + HLA-DR + macrophages in the liver tumors compared to peripheral blood monocytes [41].

The mechanisms by which the cross-talk between tumor cells and infiltrating macrophages induces upregulation of B7-H3, and how B7-H3 expression on TAMs affects their functional properties, are not fully resolved. In an *in vitro* experiment, co-culture of the monocyte cell line THP-1 with HCC cells resulted in upregulation of B7-H3 on THP-1 cells, along with differentiation into a M2-polarized phenotype in a B7-H3-dependent manner, and blocking experiments revealed a critical role of STAT3 signaling in this process [106]. In a lung cancer mouse model, in which B7-H3 was significantly elevated on TAMs compared to normal peritoneal macrophages, either supernatant from TAMs or administration of the immunosuppressive cytokine IL-10 was found to upregulate B7-H3 expression on lung cancer cell lines *in vitro* [107]. Vice versa, normal peritoneal macrophages exposed to supernatants from lung cancer cell lines had increased membrane-bound B7-H3 expression, and blockade of IL-10 partially reduced this upregulation. Thus, inflammatory cytokines such as IL-10 could be key mediators of B7-H3 upregulation on both TAMs and cancer cells in their TME.

**MDSCs** is a collective term for immature myeloid cells with either monocyte- or granulocyte-like differentiation and high phenotypic plasticity. MDSCs contribute to the immunosuppressive TME of solid cancers by inhibiting T cell and NK cell activity and suppressing anti-tumor immune responses [108]. MDSCs were found to be enriched also in pediatric solid cancers, along with both correlative and functional evidence in murine models of a key role in local immunosuppression [109–111]. Data on the presence and relevance of B7-H3 in tumor-associated MDSCs are limited. In an adult cancer, NSCLC, B7-H3 expression on MDSCs was restricted to tumor tissue, with no expression detected in MDSCs in adjacent normal lung tissue or from the peripheral blood [112]. The B7-H3-positive MDSC population was found to support tumor growth by contributing to an immunosuppressive cytokine milieu and by inducing T cells with regulatory phenotype and function. Moreover, a higher frequency of B7-H3-positive MDSCs in this study correlated with poorer survival outcomes in NSCLC patients [112].

Tumor-associated **neutrophils** can have variable functions. In selected human tumors, they were found to drive type 1 immune responses and be associated with better outcome [113], while other reports describe contributions to immune escape and tumor progression [114]. Surface expression of B7-H3 was found in a population of intratumoral CD54 + neutrophils in gastric cancer, at significantly higher density than on neutrophils in peritumoral and non-tumor tissues [115]. Functional experiments suggest a mechanism in which B7-H3 upregulation in tumor-associated neutrophils relies on GM-CSF secreted by tumor cells. In human gastric cancer patients, the frequency of B7-H3-positive neutrophils was found to increase with tumor progression and served as an independent predictor of reduced overall survival, supporting the relevance of these findings. Data in pediatric cancers are lacking.

**Fig. 3:**
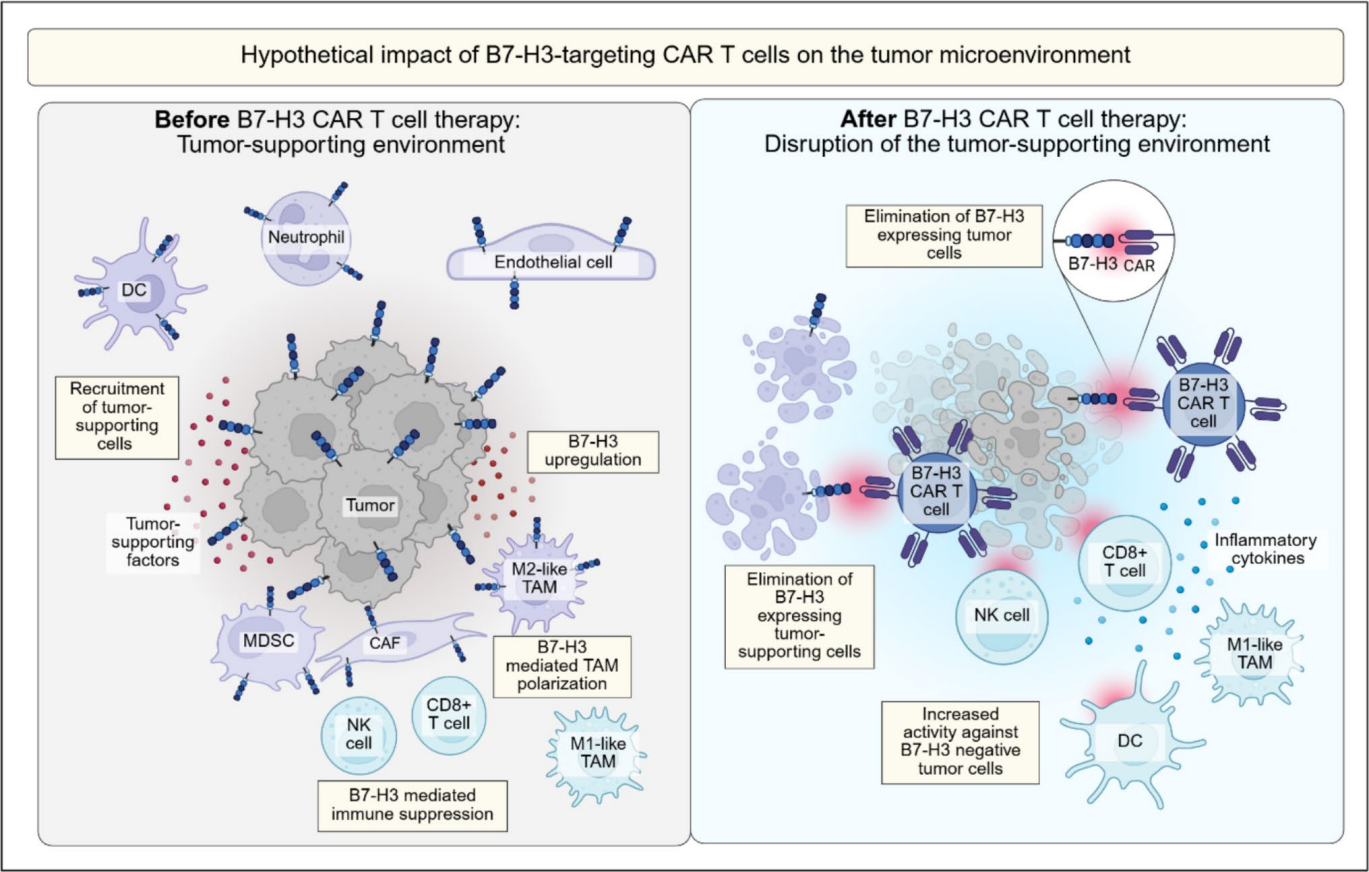
B7-H3 is expressed on tumor cells and on different cell types within the TME that support the tumor. CAR T cells targeting B7-H3 could disrupt the tumor-supporting environment and thus facilitate eradication of the tumor

Collectively, these studies support a role of B7-H3-expressing myeloid cell populations in fostering an immunosuppressive environment that supports tumor progression. Consequently, therapeutic elimination of B7-H3-positive immune cell populations could substantially alter the immune microenvironment in which tumors grow and thrive (Fig. 3).

Targeting B7-H3 with CAR T cells needs to take into account that T cells can upregulate B7-H3 protein in response to activation [81, 116, 117]. Indeed, B7-H3-specific CAR T cells were reported to express surface B7-H3 upon co-cultures with antigen-positive tumor cells. B7-H3 expression correlated with B7-H3 antigen density on the target cells [81]. Using a CAR T cell product with enhanced integrated co-stimulation via both CD28 and 4-1BB, Kristmann *et al.* observed B7-H3 upregulation and reduced *in vitro* expansion of B7-H3 CAR T cells and hypothesized that fratricide of B7-H3 CAR T cells during manufacturing could lead to suboptimal products [116]. Thus, the CAR T cell design against B7-H3 needs to be carefully selected to enable optimal antigen-induced functionality while avoiding self-limiting B7-H3 upregulation.

B7-H3 expression in tumors has also been detected on the non-immune and non-cancer cells that compose the stroma and vascular TME. Pathological angiogenesis is a hallmark of cancer [118]. The disrupted, immature vasculature of solid tumors establishes an important barrier to T cell infiltration and restricts their recruitment into tumors [119–121]. In a landmark study comparing gene expression of **endothelial cells** derived from the blood vessels of normal tissues and tumors, B7-H3 was identified as the most strikingly differentially expressed marker of tumor endothelial cells, allowing to distinguish physiological from pathological angiogenesis [122]. Subsequent research from the same group and others further confirmed the strong overexpression of B7-H3 on tumor vasculature across various adult solid cancers [54, 123–127]. A B7-H3-specific antibody–drug conjugate effectively targeted both cancer cells and tumor vasculature, resulting in eradication of large solid tumor masses in mice [128]. A single article reports consistent expression of B7-H3 on tumor-associated vasculature also in a pediatric solid cancer, osteosarcoma [77]. Together, these findings position B7-H3 as a key marker of tumor-associated, pathological vasculature across multiple tumor types and increase its attraction as a target for CAR T cell therapy. By disrupting the blood supply to tumors, co-targeting of vascular endothelial cells along with tumor cells with a single CAR T cell product could be a potent strategy to prevent escape of tumor cells with low target expression and thus overcome one of the key limitations of current CAR T cell therapeutics.

A cell population that recently has raised much interest in therapeutic co-targeting strategies are cancer-associated fibroblasts (CAFs). CAFs promote tumor progression through the secretion of ECM components, growth factors and cytokines, which collectively enhance tumor cell proliferation, survival and invasion. In addition, CAFs play a pivotal role in creating an immunosuppressive environment that prevents effective anti-tumor immune responses [129]. In syngeneic mouse models of solid cancers, CAR T cells targeting a surface marker expressed on CAFs, fibroblast activation protein (FAP), allowed effective subsequent tumor-directed CAR T cell therapy [130]. Various studies have found that CAFs in human solid tumors are B7-H3-positive. B7-H3 expression was detected on stromal fibroblasts in colorectal cancer, triple-negative breast cancer, renal cancer and gastric cancer [126, 131–133], while absent on normal fibroblasts [133]. B7-H3 positive CAFs were found to activate regulatory T cells (Tregs), promoting immune evasion [131], and tumor invasion and metastasis through the secretion of soluble growth factors [132]. Additionally, B7-H3 expression conferred an anti-apoptotic effect on the CAFs present in the TME of renal cancer [132]. Downregulation of B7-H3 in CAFs reduced the migration and invasion capacities of CAFs, underscoring its relevance in this cell population. B7-H3 knockdown in CAFs isolated from tumor tissue further resulted in decreased production of inflammatory cytokines such as IL-6 and of vascular endothelial growth factor (VEGF) [132]. VEGF is an important factor in the formation of tumor vasculature and in promoting tumor growth and metastasis, also in pediatric cancers [134, 135], and it has proinflammatory properties that overall suppress effective T cell immune responses [120, 136]. The effects on the immune system are mediated via VEGF receptors on endothelial cells and on tumor-associated myeloid cells. The finding that B7-H3-expressing CAFs contribute to VEGF production in the TME support an important role of B7-H3 in the establishment of an immunosuppressive micromilieu. Overall, the expression profile of B7-H3 on both tumor endothelial cells and CAFs makes it an attractive target for stroma-targeting strategies, with the ultimate aim to improve therapeutic immune targeting (Fig. 3).

## Therapeutic options to modulate the TME via B7-H3

**Fig. 4:**
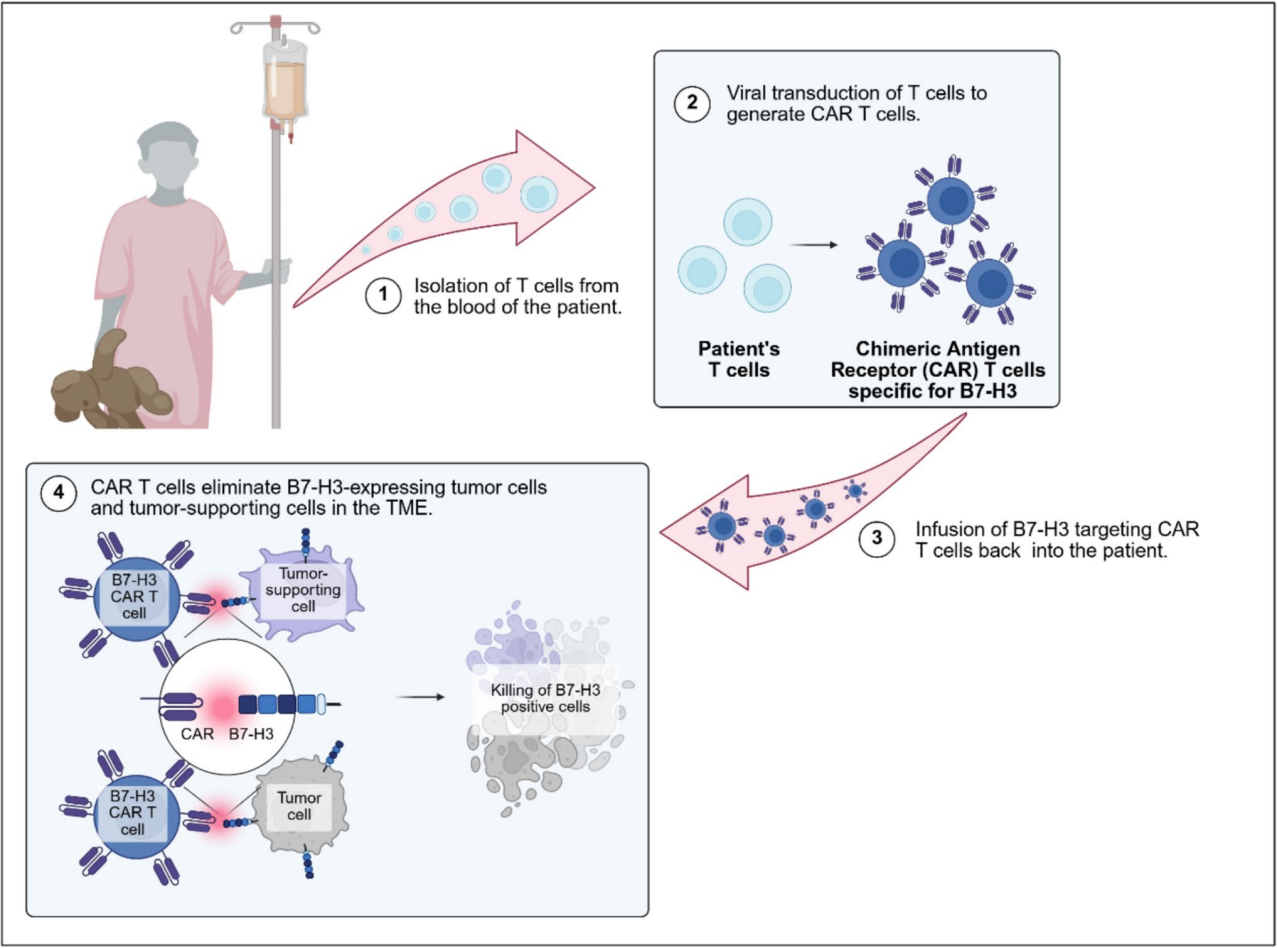
Workflow of CAR T-cell therapy, from isolation of T cells from the blood of the patient and genetic modification to administration of the CAR T-cell product back to the patient, where the CAR T cells disrupt the TME and eliminate the tumor

B7-H3 has initially been considered as a target for checkpoint inhibitors that synergize with PD-1 blockade [41]. In pediatric cancers, however, immunotherapies that rely on the activation of pre-existing T cell responses against cancer-associated neoantigens have failed, with few exceptions [137]. This is explained by the low mutational burden of most pediatric cancers [138], creating a limited repertoire of antigens recognizable as foreign, and a cold TME with scarce T cell infiltrates [139]. Preclinical *in vivo* studies have also addressed non-cellular B7-H3-targeting therapies, e.g. antibody–drug conjugates (ADCs) [26, 27, 123, 140], and generated first evidence that ADCs can simultaneously target B7-H3-expressing cancer cells, tumor-associated vasculature and CAFs. Due to their strong effector functions and capacity for antigen-induced *in vivo* expansion, CAR T cells could be an especially effective tool to eradicate both tumor cells and their supportive TME [13, 28, 58, 141–147]. For B7-H3 CAR T-cell therapy, the patient’s T cells are gene-modified *ex vivo* to express a B7-H3-targeting CAR, then the cells are infused back into the patient (Fig. 4). First evidence that B7-H3 CAR T-cell therapy affects the composition of the TME was obtained by scRNA analysis in a murine syngeneic CNS cancer model, with all the limitations of the mouse model and not addressing B7-H3 protein expression [148]. A recent study showed that B7-H3 scFv (single-chain variable fragment)-based CARs that are currently investigated for CAR T cell therapy, differ in their affinities and avidities for B7-H3 (Barisa *et al.*, Nat Commun, in press) [149], and it remains to be seen how this affects their effect on the TME. A low avidity B7-H3 binder becomes activated through high B7-H3 surface expression but not low and might only respond to tumor cells with high B7-H3 antigen density without targeting components of the TME. A high avidity binder becomes activated both through low and high B7-H3 surface expression, which could significantly modulate the TME but might raise safety concerns. The extent and manner in which B7-H3-specific CAR T cells affect the composition of solid tumors is an open question.

## Conclusions and perspectives

This review reveals a notable gap of knowledge: While B7-H3 expression on tumor cells is well-documented across both adult and various pediatric tumor types, very limited data exist on the presence and role of B7-H3 also on non-tumor cells, especially in pediatric cancers. This knowledge could be highly relevant for understanding the full mechanism of action of B7-H3 directed CAR T cell therapy and the specific challenges in the pediatric population where this treatment has first entered clinical investigation [13, 28, 29].

Based on data from various adult solid tumors, the expression profile of B7-H3 within the TME appears strongly supportive for the general rationale of B7-H3-targeted therapies. B7-H3 surface expression is found on multiple cell types that together support immune evasion, tumor persistence, metastasis and formation of the pathological tumor vasculature. Co-targeting B7-H3-expressing MDSCs and immunosuppressive TAMs as well as tumor endothelial cells and CAFs could disrupt the tumor-supportive niche and vascular networks that these cell types create and remove immunosuppressive barriers (Fig. 3). First preclinical evidence that B7-H3 targeted therapies indeed modulate the TME in the expected manner [128, 140] support the hypothesis that B7-H3 as target for CAR T cell therapy offers a unique dual advantage.

However, it is important to recognize that the TME in pediatric cancers differs significantly from that in adult tumors [139, 150]. This highlights the need for focused studies on detailed B7-H3 expression specifically in pediatric solid tumors. Such studies are essential for developing therapeutic strategies that are tailored to the distinct cellular and immune landscapes of pediatric patients. In addition, a more detailed understanding of the mechanisms and context-dependent role of B7-H3 in immune modulation is essential for developing therapies that overcome immune-suppressive effects without inadvertently hindering beneficial immune activity. Identifying all specific receptors that bind B7-H3 will be crucial for a comprehensive understanding of its function in the immune system. This consideration is especially important for pediatric patients with a developing immune system, where interventions must avoid deleterious long-term effects.

While most current insights come from preclinical studies, clinical trial results will be crucial for advancing our understanding of B7-H3 CAR T cell therapies. Results from case reports [151–153] and early clinical trials with B7-H3-targeting CAR T cells have started becoming available [13, 28, 29], offering valuable insights into the feasibility, safety, and preliminary efficacy in (pediatric) solid and CNS tumors. Despite high-density expression of B7-H3 in the targeted cancers and the encouraging, largely consistent evidence that this target marks also bystander cell populations in the TME with immunosuppressive function, first-in-human studies have not yet shown convincing antitumor activity [13, 28]. Powerful mechanisms apparently prevent adequate infiltration of B7-H3-targeted CAR T cells into tumors and their potent activation, *in vivo* expansion and antitumor functionality. Maintaining CAR T cell persistence and functionality over time also is critical. Analysis of tumor tissues obtained from patients treated with B7-H3 directed CAR T cells will be highly valuable to understand the effects of B7-targeted CAR T cells on cellular interactions in the TME and identify reasons for resistance. Specifically, comparisons of B7-H3 expression on all cell types within the tumor, with consideration of their spatial relationships, and of the cellular composition of the TME in responders and non-responders will help to understand whether and how on-target/off-tumor effects contribute to effective CAR T cell targeting of this antigen. Due to highly regulated B7-H3 protein expression (Fig. 2), gene expression analysis at single cell level can alone not answer these questions and will need to be complemented or replaced by multiplex imaging technologies.

Advances in the CAR design along with therapeutic combinations are likely needed to enable effective early activation responses that lead to elimination of B7-H3-positive tumor, stroma and suppressive immune cells and eradicate solid tumors. An additional challenge is resistance due to antigen heterogeneity with consequent outgrowth of antigen-negative clones [152, 153]. Preclinical advances include modifications of CAR designs that optimize functionality, persistence and activity in the hostile tumor environment or add an additional binder to prevent B7-H3-negative escape [143, 145, 147, 154–157]. One option are cytokine-enhanced CAR T cells that receive powerful proliferation stimuli e.g. from IL-15, as recently reported in a clinical trial with a GPC-3 specific CAR T cell product [10]. Compared to earlier patient cohorts treated with the standard CAR T cell product, IL-15 co-expression resulted in partial and even complete responses of pulmonary metastases and regression of liver manifestations, associated with substantial CAR T cell *in vivo* expansion [10]. An alternative cytokine, the inflammatory danger signal IL-18, could exert additional stimulatory effects on the CAR T cells [158] and on bystander cells in the TME [159], promoting a strong activation response while shaping T-cell supportive micromilieu. Tolerability of such combinations will require careful assessment and dose escalation, especially in the light of dose-limiting liver toxicity observed in the first responder to systemic B7-H3 CAR T cell therapy, after a second dose and associate with substantial CAR T cell expansion [13].

In summary, B7-H3 has high potential as therapeutic target for CAR T cell therapy, with widespread expression across various pediatric solid tumors. Expression of this target on diverse cell types within the TME should not be neglected, and further investigation of its functional significance is essential to develop more effective B7-H3 targeted cell therapies.

## Data Availability

No datasets were generated or analysed during the current study.
